# Being the Alice of academia: lessons from the Red Queen hypothesis

**DOI:** 10.1093/femspd/ftac034

**Published:** 2022-09-14

**Authors:** S G Negatu, M C Arreguin, K A Jurado, C Vazquez

**Affiliations:** Department of Microbiology, University of Pennsylvania Perelman School of Medicine, 451B Stemmler Hall, 3450 Hamilton Walk, Philadelphia, PA 19104, United States; Department of Microbiology, University of Pennsylvania Perelman School of Medicine, 451B Stemmler Hall, 3450 Hamilton Walk, Philadelphia, PA 19104, United States; Department of Microbiology, University of Pennsylvania Perelman School of Medicine, 451B Stemmler Hall, 3450 Hamilton Walk, Philadelphia, PA 19104, United States; Department of Microbiology, University of Pennsylvania Perelman School of Medicine, 451B Stemmler Hall, 3450 Hamilton Walk, Philadelphia, PA 19104, United States

**Keywords:** underrepresented scientist, Red Queen hypothesis, adaptation, academia

## Abstract

Viruses and hosts must navigate environments in which each tries to outcompete the other for survival or to coexist within the same spaces. In Lewis Carrol’s *Through the Looking Glass*, the Red Queen tells Alice, “Now, here, you see, it takes all the running you can do, to keep in the same place. If you want to get somewhere else, you must run at least twice as fast as that!” Borrowing from this idea, the Red Queen hypothesis asserts that organisms, such as viruses, must continuously adapt to environmental pressures to survive. In this commentary, we draw parallels between the Red Queen hypothesis and the experiences scientists of color navigate to thrive in academic spaces. In both phenomena, adapting to environmental pressures is necessary for survival. We identify the various pressures and bottlenecks faced by historically underrepresented groups in academia, as well as the adaptation strategies they must implement to persist in academia.

## Teaser

The authors draw parallels between the microbiological concept of the Red Queen hypothesis, which asserts that both pathogens and hosts must adapt to evolutionary pressures, with the experiences of underrepresented groups in the sciences.

## Introduction

Consider working hard with the intent of propelling your career in the academic world of biomedical sciences and only obtaining incremental progress, much like Alice in *Through the Looking Glass* as she tried to outrun the Red Queen. In the novel, the Red Queen suggested that Alice was running to stay in the same place, and it was necessary that Alice run a minimum of twice as fast to get anywhere. This very concept was adapted by Dr Leigh van Valen, an evolutionary biologist, to generate the Red Queen hypothesis. He posited that species must adapt or run the risk of becoming extinct. The Red Queen hypothesis is commonly accepted today to highlight the evolutionary arms race between pathogens and hosts. To gain an advantage over the other, pathogens must continuously adapt to pressures placed on them by our immune systems; likewise, our immune systems must mount countermeasures to prevent pathogen persistence. Alice’s experience and the constant adaptation of species to survive is the reality of many underrepresented scientists in challenging academic environments.

The Red Queen hypothesis suggests for pathogens and hosts to simultaneously persist, they must continuously change. Viruses undergo evolutionary pressures that include, but are not limited to: differential hosts, immune responses, and/or antiviral pharmaceuticals (Frost et al. 2018). Viruses mount evolutionary pressures against humans by introducing viral immune evasion mechanisms and novel cell entry and replication strategies. This coevolution is an iterative process that takes hundreds, or even thousands, of years. In academia, there are similar selective pressures that underrepresented scientists, particularly women of color, experience. The experiences of underrepresented women in science are impacted by the intersection of multiple identities (i.e. race and sex), further exacerbating the challenges faced by this group. Due to limited studies focused on women in science specifically, we aim to discuss the academic pressures, required assimilations, and academic bottlenecks faced by underrepresented scientists overall in this commentary. The following section will discuss the academic pressures that result in what is been coined the “leaky pipeline” (Hinton et al. 2020).

## Academic pressures toward underrepresented scientists continue to encourage systemic exclusions in academia

Social media has revolutionized the challenges underrepresented academics face and highlighted that these problems exist nationwide. #BlackInTheIvory, a social media-driven movement, has made invisible Black academics visible by amplifying the voices of these trainees (Subbaraman 2020). Stories highlight the day-to-day challenges Black trainees in academia face. Many underrepresented scientists often feel unwelcomed at the institutions they belong to. For example, using the #BlackInTheIvory platform, an individual shared their reality of wearing university gear as a necessity to communicate to others on campus that they are not a threat. Another student anonymously shared that they were followed by campus police following a late-night study session at the campus library: “I was followed by campus police for ‘trespassing’ my own campus upon leaving the library on a late night after studying. There were others who left the same time I did. None of them were Black. No one else was questioned. Just me” [*Black Ivy Stories (@blackivystories) • Instagram Photos and Videos*, Black Ivy Stories 2022]. Being questioned about whether one is a member of the university is unwelcoming, and a common academic pressure experienced by underrepresented trainees at universities across the country. These experiences highlight that majority faculty, trainees, and staff are not only unable to see where underrepresented students fit in at the university, but assumptions are also made for what the role of the underrepresented person may be.

Beyond attempting to make it physically apparent that people of color are a part of university communities, underrepresented scientists strive to prove their presence at institutions is valuable enough to be taken seriously. Henry Henderson, a cancer biology postdoc at the Vanderbilt University Medical Center, describes his experience of presenting a poster as a first author at a national conference, and being questioned as to who did the work that was being presented (Gewin 2020). Despite these negative, unwelcoming experiences, Black academics continue to work hard, ask great scientific questions, and execute well-thought-out experiments. Rather than questioning the capabilities of Black scientists, why not invite underrepresented academics to the table for authorship, presentations, and collaborations?

In 2020, Hofstra and colleagues revealed that underrepresented US PhD recipients from three decades were more innovative compared to the majority, yet their novel contributions were less likely to aid in obtaining academic positions (Hofstra et al. 2020). This diversity–innovation paradox highlights the challenges underrepresented scientists aspiring faculty positions face. Although diversity breeds innovation, and innovation supposedly leads to successful careers, this is not guaranteed for scientists of color due to academic pressures such as exclusive environments and varying standards.

An academic pressure, i.e. not often considered is that in both graduate school and postdoctoral training, stipends and salaries are often not enough to cover several of the financial costs these positions accrue. For example, there are the upfront costs of traveling to a city for research experience and being paid at the end of the first month, or worse at the end of the summer. Graduate students also encounter similar problems when first beginning PhD programs, as many stipends are provided at the end of the first month or later. Further, in many institutions, trainees must pay up front for conference registration, housing, and travel. Low salary and stipend levels may also disproportionately affect trainees of color. In a commentary published in *Molecular Biology of the Cell*, Andrade and colleagues highlighted that the COVID-19 pandemic exacerbated the already fragile financial reality many underrepresented scientists face. They outline several factors contributing to this disparity, including lack of generational wealth and caregiving responsibilities, as many postdocs of color do not have financial support from family members but instead often provide that financial support for their immediate in addition to their own financial needs (Andrade et al. 2022). In the same commentary, they also provide an example of an IRACDA postdoc at San Diego who shared that “30% of his total income goes to provide care for his aging parents, and many fellows are the primary caregivers for both their immediate and extended families.” Financial limitations are inadequately documented; thus, more studies are needed to evaluate both the availability and feasibility of undergraduate, postbaccalaureate, and graduate research opportunities that are intended to improve diversity and inclusion in science.

Faculty academic pressures on the other hand are much better documented. Gender biases in NIH funding, publishing, and ultimately tenure determination have been discussed in several manuscripts. A 2018 study looked at the NIH funding disparities by gender and found women held fewer grants and were less successful in renewing grants, compared to men (Hechtman et al. 2018). Additionally, manuscript rejection has also been found higher amongst women (Fox and Paine 2019). These systemic gender biases require women to work harder than male counterparts. In addition to these challenges, women of color face further pressures as they also face race biases. Recent publications have brought attention to the origins and challenges of racial inequality within academia (Dupree and Boykin 2021). Overall, underrepresented faculty are limited in number and encounter constant instances of racial ignorance (or color blindness) and stereotype threat. Furthermore, these data reveal that women of color scientists face increased pressures due to intersecting identities (i.e. race and gender). Although more studies are needed to explicitly study the experiences of women of color in science, the limited number of faculty with these identities poses the challenge of conducting studies while maintaining anonymity.

The need for underrepresented scientists to continue to adapt to academic pressures does not end once they attain a tenure-track faculty position, as the threat of not being granted tenure looms in the background. Several stories of faculty of color being denied tenure have recently made headlines. Dr Nikole Hannah-Jones is the inaugural Knight Chair in Race and Journalism at Howard University, a MacArthur Genius Fellow, a Pulitzer Prize-winning journalist, and author of the widely contested *1619 Project*. Prior to her appointment at the Howard University, Dr Hannah-Jones received full endorsement and tenure recommendation by The University of North Carolina (UNC) at the Chapel Hill’s journalism department, but was denied tenure by the Board of Trustees (though after the public reprimand received by UNC, they offered her tenure, which she denied for Howard; Robertson 2021). The likely reason for the tenure dismissal was her book the *1619 Project*, which recounts the original beginnings of slavery in the US. If a Pulitzer Prize-winning journalist was denied tenure, what does that say about the prospects of other underrepresented faculty earning tenure? More recently, in February 2022, Dr Michael W. Kraus, an Associate professor at the Yale School of Management who studies inequality, was denied tenure even though his research has been cited over 10 000 times [*SOM Tenure Denial Sparks Debate on Diversity in Academia*(Yale Daily News 2022)]. These tenure examples illuminate a larger problem within academia of the extra barriers faculty of color must overcome to potentially reach their academic goals.

Evolutionary pressures result in both pathogens and hosts to either evolve over time to survive, or not survive and, therefore, leave the population. In the academic setting, underrepresented trainees either assimilate to remain on track for an academic faculty position, or individuals leave the pipeline and no longer pursue the world of academia. In this next section, we will delve into initial assimilations of trainees when entering academia.

## Academics of color must assimilate to succeed in the current academic climate

SARS-CoV-2 has recently taught us that to persist in the population, multiple mutations are necessary. Similarly, academics of color must also adapt to persist in academia. Code switching, which entails shifting the way one expresses oneself in conversation to fit in (Thompson 2013), is one example of a common assimilation. Dr Mariana Viglino, a postdoctoral fellow at the National Scientific and Technical Research Council’s Patagonian Institute of Geology and Paleontology, and Ana Valenzuela-Toro, a PhD candidate at the University of California, Santa Cruz, have discussed some assimilations that they experienced during their time in academia. They mention that there are numerous “hidden or unwritten norms, ranging from dress codes (what does ‘business casual’ even mean?), to how to navigate social events and pitch oneself and research work to colleagues or potential mentors.” From their perspective, women of color scientists must assimilate to perform at a higher level than their colleagues (Valenzuela-Toro and Viglino 2021).

Assimilating to be a professional leads to slowly losing personal cultural identities (Morales 2021), while academic environments are unaware of the efforts done to assimilate to academic culture. Some of the consequences of assimilation for trainees of color in science are well-represented in Dr Jennifer Morton’s description of the sacrifices endured by “strivers,” who she defines as low-income or first-generation college students. Both groups suffer the ethical costs of upward mobility, which include “relationships with family and friends, connection to one’s community, and one’s sense of identity,” all of which give value and meaning to lives (Morton 2019). The summation of the different assimilations not only leads underrepresented scientists feeling alienated from their own communities, but the constant act of assimilating takes mental resources that could be dedicated to academic work instead (Morales 2021). One study found that decreased contact with heritage and increased depression were predicted by an increase in assimilation (Lechuga and Fernandez 2011). From this, women of color in science learn that to succeed in academia, they must make changes to fit into the new environment, just like pathogens adapt in response to pressure.

The expectation to perform at higher levels than their majority-represented counterpart’s results in underrepresented groups working twice as hard to succeed in academia, which is another assimilation often adopted. One study specifically examining the perception of sexual harassment, sexual assault, and women’s leadership at a research university reported that women perceive that they are being held to a higher standard and expected to “be twice as good and work twice as hard” (Evans et al. 2019). Even before encountering academic spaces, many Black workers have experienced familial pressures suggesting that to succeed, despite racial discrimination, they need to be: “twice as smart, twice as dependable, twice as talented” (White 2015). The experience of Dr Chrystal Ama Starbird, during her postdoctoral tenure at the Yale University, demonstrates that even working twice as hard, enduring many personal challenges while still excelling in her graduate studies, can still not be enough. In her second year as a graduate student, she was at the top of her class and despite the class consisting of only 10 people, the professor actively confused her with the least performing student in the class. Even at the end of the semester, her efforts and exceptional work went unnoticed, as she describes “it was clear that scholarly discourse was never to be had between this professor and me.” (Starbird 2021). Her experience is a clear example that assimilating to the culture of academia and excelling is not enough to be taken seriously as a scientist.

Immunology and adaptive immunity have taught us that diversity is not only helpful, but integral to ensure protection against endless encounters with pathogens—integration is contradictory. Indeed, underrepresented scientists recognize that even postassimilation, we are not the same as other colleagues, so why assimilate at all (Morales 2021)? Including more underrepresented scientists increases our diversity and creativity, which can be lost by expecting assimilation (Morales 2021). Alas, attempts to “fit in” with existing cultures are not sufficient. Academia must recognize that diversity and excellence are mutually reinforcing rather than mutually exclusive (Evans et al. 2019). There is evidence that more scientific productivity comes from having diverse research groups (Freeman and Huang 2014, AlShebli et al. 2018, Powell 2018). Perhaps we need a new definition for professionalism, one that includes a culture of mutual respect (Morales 2021), tolerance, and more precise communication (Powell 2018). Ensuring an inclusive community is only one step—the next section discusses bottlenecks present in academia.

## Academia bottlenecks impede the success of underrepresented scientists

Pathogens and hosts have evolved strategies for survival. Yet, there are events, or bottlenecks, threatening the replicative success of each. Similar obstacles are in place in academia. The existing architecture within academia precludes individuals from underrepresented groups from even entering academia. Initially pursuing a scientific career is difficult: from lack of representation of scientific role models while growing up to the accepted criteria to uproot one’s family and leave behind familiar communities. This represents the first bottleneck scientists of color must face to even approach an academic career. In this section, we aim to highlight the academic bottlenecks that impede the success of underrepresented groups once they have entered the academic research spaces.

A recent PEW study found that Hispanic employees make up 8% of the STEM workforce, while Black employees comprise 9% of the STEM workforce (Funk and Parker 2018). R01s, the currency of tenure determination, do not paint a better picture. R01s from White scientists are funded at approximately double the rate of those from Black scientists. In the 2020 fiscal year, 5% of R01 awardees from the National Cancer Institute were LatinX PIs and 1% were Black PIs, compared to 64% of White PIs (Taffe and Gilpin 2021). Academia and STEM fields cannot continue with this current disparate funding climate. Given the current tight bottleneck, more scientists of color will continue to leave academia, further contributing to the “leaky pipeline” (Hinton et al. 2020). This leads to another bottleneck: the dependence of funding on obtaining a postdoc position, faculty position, and tenure. Throughout the academic pipeline (graduate student–postdoc–faculty), obtaining funding is presumed to be an indicator of future research success. Earning a fellowship in graduate school may lead to another fellowship down the line, which may lead to a competitive NIH grant and tenure-track faculty position. The NIH makes concerted efforts to fund underrepresented scientists at early stages of training, yet few mechanisms exist for faculty. Fellowships such as the Howard Hughes Medical Institute Hanna H. Gray and Freeman Hrabowski fellowships and the Chan Zuckerberg Science Diversity Leadership Award aim to reduce this bottleneck, but more work remains to address the gap in research funding where clear disparities exist. Within the R01 application process at the NIH, Black scientists obtain a mere half the rate of funding as compared with White scientists (Hoppe et al. 2019). R01 applications from Black scientists are less likely to be discussed and they receive lower impact scores (Hoppe et al. 2019). Interestingly, topic choice reduced this funding gap by 21% (Hoppe et al. 2019). Given this stark reviewer preference for certain topics, more intentional efforts are needed to ensure reviewer pools are diverse and representative. Recent data indicate that reviewers are more likely to be men (61.1%) than women (38.9%) (Volerman et al. 2021); intersectionality not being considered underscores the importance of this facet in future funding reviews and reviewer selection.

Faculty from underrepresented groups disproportionately perform community and outreach service. Collectively, this is termed the “minority tax,” a toll placed on underrepresented faculty to achieve equity and diversity within academic spaces (Rodríguez et al. 2015, Trejo 2020). This tax also trickles down to trainees of color, as more students and postdocs of color lead diversity, equity, and inclusion (DEI) initiatives in their departments or institutions, compared to their white colleagues (Williamson et al. 2021). This is evident in the numerus DEI initiatives that arose from the murders of Ahmaud Aubery, George Floyd, and Breonna Taylor in 2020. Since then, many universities have launched DEI groups, including Cornell University, the University of Minnesota, and others (*How George Floyd’s Death Changed College Campuses*, TheBestSchools.Org^2021^). These efforts are noteworthy and underscore the burden placed on underrepresented trainees to spearhead and lead these initiatives; however, this work alone does not sufficiently feed into progress in academia and requires input from institutional overhead, those in leadership positions, and White colleagues.

Academic progress can stall for underrepresented scientists as they try to balance research with community engagement and diversity efforts, thus requiring them to do more work to prevent this block in progress. This is not to say that underrepresented scientists do not enjoy or place an importance on such initiatives, as many do. Yet, they are also academic scientists, with pressure to produce publications and grants to get a PhD within the recommended time frame or earn tenure, which are many of their primary responsibilities. How then does one balance all these responsibilities when it seems as though universities do not recognize or reward DEI efforts on par with research efforts?

In addition to the consequence, this extra labor places on academic progress, there is an emotional burden placed on underrepresented scientists, referred to as the “emotional tax” [“Understanding the Emotional Tax on Black Professionals in the Workplace” (Bloomberg LP 2022)]. Dr Emma Hernandez-Sanabria, a senior postdoctoral fellow at the KIB in Belgium, says it perfectly: “minorities have traditionally resorted to develop a ‘thick skin,’ reinventing career paths and paving the way for new generations” (Hernandez-Sanabria 2020). As was first mentioned in this section, one of the first bottlenecks experienced by scientists of color is having to leave behind their family, loved ones, community, and sense of belonging to pursue an academic career. This places a huge emotional toll on those scientists as they must then navigate the inherent struggles of graduate school plus the struggles of being one of the few scientists of color at their respective institutions and in academia overall. The emotional tax also includes being unable to establish a core group of confidantes for advice or to navigate potentially difficult situations or to seek out mentors as there are so few faculty of color and peers of color. Recently, more emphasis and awareness has been placed on the mental health of biomedical students and faculty, especially as the world deals with the current COVID-19 pandemic, likely already enhancing the already emotional turmoil of underrepresented trainees (Dukes 2020).

Black students must also deal with an increased toll on their mental health, while also navigating the other bottlenecks discussed above. During a panel on the mental health impacts of racism and discrimination at the Harvard T.H. Chan School of Public Health in 2019, Dr John Silvanus Wilson stated that “students of color are less aware of mental health services, less likely to be diagnosed, and less likely to be treated” (Powell 2019). He also emphasized the importance of institutions earning Black students’ trust and creating a more welcoming environment to help ease the mental health burden they face. Underrepresented faculty are not immune to this emotional tax either. Underrepresented faculty also report feeling anxious and an increased sense of not belonging (Zambrana et al. 2021). Collectively, these experiences highlight the need for institutions to work on establishing trust with underrepresented scientists, make mental health services more accessible and advertised, and promote belonging and a sense of a community among faculty of color. This goal can be achieved by cluster hires, which the NIH is currently trying to implement [*NIH’s New Cluster Hiring Program Aims to Help Schools Attract Diverse Faculty* (NIH 2022)].

Many faculty of color serve as shadow mentors to trainees of color, further adding to their growing list of obligations (Davis-Reyes et al. 2022). For many underrepresented scientists, there exists an obligation to accept these extra roles because who else would serve? Underrepresented scientists also see versions of their younger selves who searched for role models that looked like them yet were few and far between. A common thread in many graduate school and faculty applications is a diversity statement, where the university asks the prospective applicant how they contribute to diversity or diversity initiatives. While this is likely meant with good intent to improve representation in academia, this adds to the emotional tax on scientists of color as individuals must relive and share pain and trauma to unfamiliar people (*How Applying to Grad School Becomes a Display of Trauma for People of Color*, Electric Literature 2018).

## Final remarks

**Figure 1.**
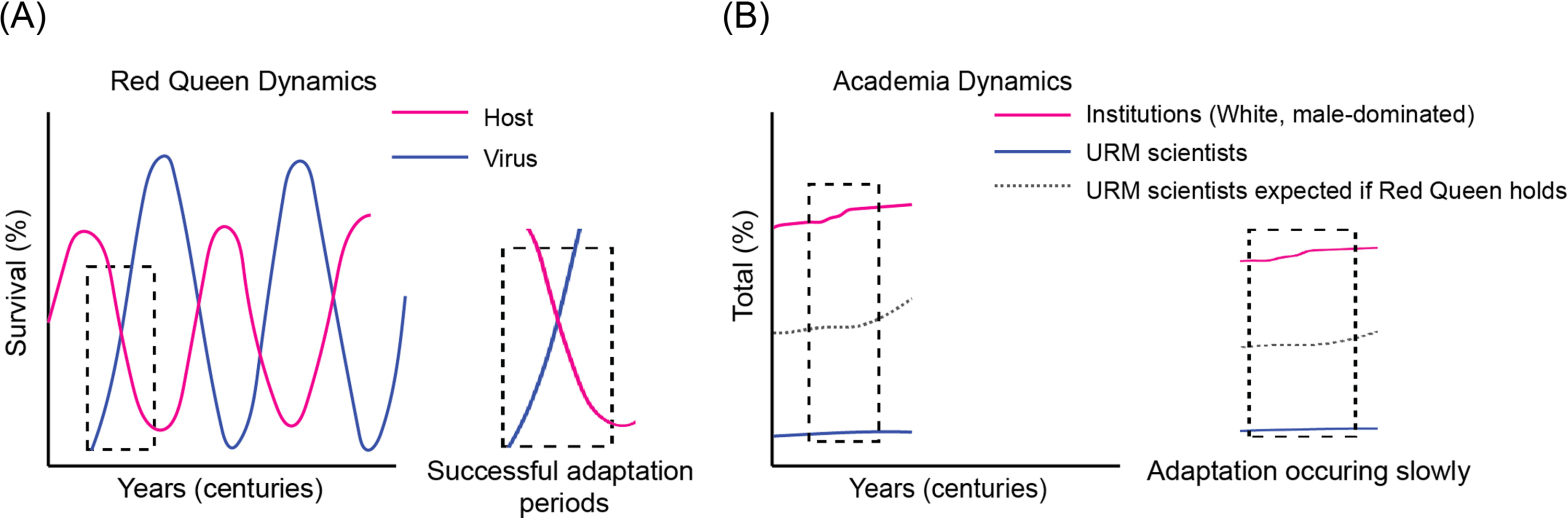
Comparison of theoretical Red Queen dynamics and academia dynamics. (A) Theoretical representation of Red Queen dynamics with the dotted box indicating events where successful viral adaption has occurred over centuries of evolution [informed from Gibson et al. (2015), Strotz et al. (2018)]. (B) Hypothetical data from representative trends of underrepresented biomedical scientists in receiving NIH support or faculty positions (Bennett et al. 2020, NIH Data Book 2022b). Dashed box highlights that successful adaptation events have not occurred.

In this commentary, we aim to highlight the main commonality between academia and the Red Queen hypothesis: adaptation as a means of survival. Over centuries, viruses and humans continue to evolve by introducing oscillating adaptions as means for existence, with periods of successful adaptations where the frequency of host or virus supersedes the other (Fig. 1A). In academia, however, underrepresented scientists attempt to bring rapid cultural changes in a much shorter period, on the scale of decades. Yet, based on data reports available on the NIH website (NIH Data Book 2022a, 2022b, 2022c), the number of underrepresented groups (African American, Hispanic, and American Indian/Alaska Native) that are PhD recipients, principal investigators, and graduate students in the biomedical sciences remain much lower (at least 10-fold) than their White colleagues, and this disparity does not appear to have changed over the last 20 years [Fig. 1B; adapted from Bennett et al. (2020), NIH Data Book (2022b)]. Currently, we are far from reaching a successful adaptation event where minorities in science can thrive within the current academic structure. Though we highlight the struggles and adaptations scientists of color endure to adapt, we would like to emphasize that eventually our aim is to diverge from the Red Queen hypothesis. We would like successful adaptation to occur, and once academic spaces reach equilibrium (equity and equality), then academia will thrive.

Here, we discussed the adaptation mechanisms employed by scientists of color, i.e. assimilations. Moving forward, it is important for the field to not confuse these assimilations with “professionalism.” As the academic field becomes more diverse, we expect to see a cultural shift that redefines “professional” compared to initial standards that were defined at a time when the field was entirely White male scientists. While the recruiting efforts of DEI initiatives are much appreciated, it is critical that discussions about the retainment of diverse scholars are employed. Retention and support of underrepresented scientists within academia are fundamental to their success and for shifting the academic culture. It is not just about admitting trainees of color into academic positions and then expecting them to change to fit in, but to adapt the culture of academia where they do not lose themselves in the process. In conclusion, NIH data about PhD recipients and faculty grant renewal stratified by race/ethnicity suggest progress has been relatively stagnant. We recognize that our contributions as women of color in academia, or our “minority taxes,” are for the betterment of the academic society, it is critical majority scientists are helping the invisible feel visible as this is the only path to change.
